# In Vivo Effects of Einkorn Wheat (*Triticum monococcum*) Bread on the Intestinal Microbiota, Metabolome, and on the Glycemic and Insulinemic Response in the Pig Model

**DOI:** 10.3390/nu11010016

**Published:** 2018-12-20

**Authors:** Francesca Barone, Luca Laghi, Andrea Gianotti, Domenico Ventrella, Danielle Laure Taneyo Saa, Alessandra Bordoni, Monica Forni, Patrizia Brigidi, Maria Laura Bacci, Silvia Turroni

**Affiliations:** 1 Department of Veterinary Medical Science, University of Bologna, 40064 Ozzano dell’Emilia, Italy; francesca.barone7@unibo.it (F.B.); domenico.ventrella2@unibo.it (D.V.); monica.forni@unibo.it (M.F.); marialaura.bacci@unibo.it (M.L.B.); 2 Department of Agro-Food Science and Technology, University of Bologna, 47521 Cesena, Italy; l.laghi@unibo.it (L.L.); danielle.taneyosaa2@unibo.it (D.L.T.S.); alessandra.bordoni@unibo.it (A.B.); 3 Department of Pharmacy and Biotechnology, University of Bologna, 40126 Bologna, Italy; patrizia.brigidi@unibo.it (P.B.); silvia.turroni@unibo.it (S.T.)

**Keywords:** einkorn, gut microbiota, gut metabolites, glycemic response, insulinemic response

## Abstract

Einkorn wheat (*Triticum monococcum*) is characterized by high content of proteins, bioactive compounds, such as polyunsaturated fatty acids, fructans, tocols, carotenoids, alkylresorcinols, and phytosterols, and lower *α*-, *β*-amylase and lipoxygenase activities compared to polyploid wheat. These features make einkorn flour a good candidate to provide healthier foods. In the present study, we investigated the effects of einkorn bread (EB) on the intestinal physiology and metabolism of the pig model by characterizing the glycemic and insulinemic response, and the microbiota and metabolome profiles. Sixteen commercial hybrid pigs were enrolled in the study; four pigs were used to characterize postprandial glycemic and insulinemic responses and twelve pigs underwent a 30-day dietary intervention to assess microbiota and metabolome changes after EB or standard wheat bread (WB) consumption. The postprandial insulin rise after an EB meal was characterized by a lower absolute level, and, as also observed for glucose, by a biphasic shape in contrast to that in response to a WB meal. The consumption of EB led to enrichment in short-chain fatty acid producers (e.g., *Blautia*, *Faecalibacterium,* and *Oscillospira*) in the gut microbiota and to higher metabolic diversity with lower content of succinate, probably related to improved absorption and therefore promoting intestinal gluconeogenesis. The observed changes, at both a compositional and metabolic scale, strongly suggest that EB consumption may support a health-promoting configuration of the intestinal ecosystem.

## 1. Introduction

Einkorn wheat (*Triticum monococcum*) was one of the first crops domesticated approximately 12,000 years ago in the Near East, alongside emmer wheat (*Triticum dicoccum*). Typically, einkorn was cultivated on marginal agricultural land, being able to survive in harsh environments and poor soils where other types of wheat could not survive. Spelt wheat (*Triticum spelta*) represents a hexaploid series of the *Triticum* genome constitution, which is characterized by great adaptation to a wider range of environments [1]. When compared to polyploid wheats, it has a higher content of proteins and some well recognised bioactive compounds, such as polyunsaturated fatty acids, fructans, tocols, carotenoids, alkylresorcinols, phytosterols, and lower *α*-, *β* -amylase and lipoxygenase activities [2]. These compositional traits make einkorn flour a good candidate to provide healthier foods. Specifically, the presence of antioxidant compounds and the protein profile are expected to be related to reduced cardiovascular disease and hypoallergenic effects, respectively. In particular, einkorn was shown to express few T-cell stimulatory gluten peptides, with important implications for celiac disease [3]. In vitro digested einkorn breads evidenced their higher carotenoid level as compared to modern wheats and showed a greater anti-inflammatory effect than the control (wheat bread) in Caco-2 intestinal epithelial cells [4]. Given the crucial role of the gut microbiota in the metabolism of dietary compounds, including the bio-activation of plant polyphenols into health-promoting metabolites and the production of short-chain fatty acids (SCFAs, mainly acetate, propionate, and butyrate) from fiber fermentation, as major orchestrators of the host physiology [5], it is also reasonable to speculate similar health benefits of einkorn in vivo. However, despite a big debate over the last few years on the realistic health potential for humans of the ancient grain landraces cultivated over the world, to date, only a few studies have investigated their in vivo impact [1,6]. Specifically, for einkorn, one of the most representative ancient grains, in vitro results evidenced a good healthy potential because of its effects on blood concentrations of glucose and insulin with a view to using einkorn-based foods in metabolic diseases [7,8], but none has considered changes in the microbiota structure as well as in the intestinal repertoire of metabolites, potentially influencing multiple metabolic and immunological pathways that are relevant to host health. In an attempt to bridge this gap, here we explored the gut microbiota and metabolome of pigs fed with an einkorn versus wheat-based bread. Pigs have significant anatomical and physiological similarities with humans, particularly with regard to the intestinal structure, with comparable transit time and analogous digestive and absorptive processes [9,10]. Furthermore, like humans, they are true omnivores, unlike other potential mammalian models, such as dogs, cats, ruminants, rabbits, and rodents, which have evolutionarily developed alternative digestive strategies. Finally, both pigs and humans are colon fermenters and have similar colonic microbiota composition. All of these features make the pig one of the most important models in the field of nutrition [11,12]. Through the pig model, in the present study we investigated the impact of a 30-day nutritional intervention with einkorn or wheat bread on the intestinal ecosystem, by means of next-generation sequencing of the 16S rRNA gene and metabolomics of fecal samples, as well as samples from ileal and colonic compartments. The effects of einkorn vs. wheat bread on animal physiology, blood parameters, postprandial glycemia, and insulin response were also evaluated.

## 2. Materials and Methods

### 2.1. Experimental Breads

The experimental breads that were used in this study were prepared by the bakery company Vini, Poland. Two types of breads were produced with standard white wheat and einkorn flour, respectively. They were leavened by baker’s yeast (2.5% flour basis) at 30 °C for 1.5 h and then baked at 195 °C for 45 min to obtain standard wheat and einkorn bread (WB and EB, respectively). WB and EB were dehydrated and grinded and were then delivered to the Department of Veterinary Medicine of Bologna University to feed experimental animals.

### 2.2. Animals and Study Design

All of the activities performed in this study were regulated by protocols that were approved by the Italian Ministry of Health with D.Lgs n.116/92 and were in accordance with the local guidelines for the use of laboratory animals. The study was divided in two trials: trial 1 (Tr1) that aimed to evaluate glycemic and insulinemic postprandial responses to WB and EB; and, trial 2 (Tr2) that aimed to evaluate intestinal and fecal microbiota and metabolome composition after 30 days of WB or EB dietary trial. The animals, which were purchased at a local farm, were delivered to the Department of Veterinary Medicine of Bologna University, five days (acclimation period) before the beginning of the experiments. The pigs were all fed with Swine Standard Diet (SSD) during the acclimation period. The standard diet was particularly formulated for pigs between 30 and 80 kg by CESAC s.c.a. Conselice (Ravenna, Italy). The diet composition is reported in Supplementary Table S1.

In Tr1, four commercial hybrid pigs Landrace x Duroc (females of 50 ± 2 kg, fourteen weeks old) were surgically cannulated in the jugular vein with a central permanent silicon catheter (BBraun, Bethlehem, PA, USA) under general anesthesia and stabled in single overhead boxes. For the surgery, the pigs were premedicated with an intramuscular (IM) bolus of ketamine (20 mg/kg) and medetomidine (0.03 mg/kg). Ten minutes after, when recumbent, venous access was achieved through an auricular vein and the animals were inducted with a bolus of thiopental (0.25 g/animal). The same venous access was used for fluid therapy (Lacteted Ringer, 10 mL/kg/h) and was removed at the end of the surgical procedure. Orotracheal intubation was performed using an 8-mm internal diameter (ID) endotracheal tube. Anesthesia was maintained with isoflurane (2–3%) in a 1:1 mixture of oxygen and air. After a week of recovery, the animals were trained to eat 50 g of experimental bread within five minutes. Training sessions were held at 8 a.m. in the morning, after 15 h of fasting for seven consecutive days. At the end of training period (seven days), in the morning, after 15 h of fasting, pigs were fed with 50 g of experimental bread or 50 g of glucose (liquid solution) as control food (C). Each animal was assigned randomly to a diet (WB, EB, or C) for nine days in order to replicate each diet three times. Blood samples were collected from jugular vein 15 min before the administration of the experimental bread and every 15 min for 2 h to measure glucose and insulin levels.

In Tr2, twelve pigs (six neutered males M and six females F of 36 ± 4 kg, ten weeks old) were randomly divided in four boxes (three animals each box) according to their own gender. For 30 days, the animals were fed twice a day with a 1:1 mixture of experimental bread and SSD, two boxes (n = 6; 3 M and 3 F) with WB and two boxes (n = 6; 3 M and 3 F) with EB. The animals received 1 kg each of mix diet divided in two separated meals, water was served *ad libitum*. The animals were weighed at 0, 7, 14, and 30 days; a score was daily assigned to each box to classify the feces: 0 for normal feces; 0.5 for fluffy feces; and one for watery faces. Fecal samples were collected for each animal as described in sampling session at 0, 15, and 30 days for gut microbiota evaluation and to investigate differences among digestive processes by the NMR and gas chromatography-mass spectrometry (GC-MS) technique. Blood samples (in K3-EDTA tubes) were collected at the beginning and at the end of the trial for clinical evaluation (complete blood count–CBC).

### 2.3. Sampling and Blood Analysis

In Tr1, the blood sampled from the jugular catheter was immediately analyzed by rapid test glucometer (Contour Glucometer Xt, Bayer S.p.a., Leverkusen, Germany) for the blood glucose detection while the blood for the insulin quantification was collected in a K3EDTA tube and stored at +4 °C until the end of the postprandial sampling period. The K3EDTA tubes were then centrifuged (1000 *g* × 10 min) and the plasma was stocked at −20 °C. The insulin level was detected from plasma with a chemiluminescent immunoassay at the Veterinary Clinical Pathology Service of DIMEVET (UNIBO). The area under the curve (AUC) was calculated and then glycemic and insulinemic index (GI and II) for each diet were calculated by the following proportion [GI and II = AUC food/mean (AUC reference) × 100] [13]. Dietary glycemic and insulinemic load (GL and IL) were calculated taking into account the amount of carbohydrate for each bread [GL and IL = (GI or II × g of available carbohydrate)/100] [14]. In Tr2, blood samples at the beginning and at the end of the trial were collected from jugular vein under general anesthesia with the following protocol: intramuscular (IM) administration of azaperone (2 mg/kg), after 10 min in dark and quite condition an IM bolus of ketamine (40 mg/kg). Venosafe*Q*R plastic tubes for K3EDTA (Terumo, Tokyo, Japan) were used for blood sampling, the samples were analyzed at the Veterinary Clinical Pathology Service of DIMEVET (UNIBO) for complete blood count (CBC). For fecal sampling, an operator was standing in the box with the animals. The samples were collected directly when spontaneous defecation occurred before contamination and immediately stored at −20 °C for 24 h. Samples were then stored at −80 °C until the moment of analysis. At the end of the trial, the animals were euthanized with 0.3 mL/kg Tanax*Q*R (MSD Animal Health Italia, Milano, Italy) under general anesthesia for the sampling of GI content and mucosae (colon and small intestine). Briefly, the digestive tract was removed and separated into its anatomical parts, scraping of the ileum and colon mucosae was performed, as previously described [12].

### 2.4. Microbial DNA Extraction, Illumina MiSeq Sequencing and Data Processing

Total bacterial DNA was extracted from feces and ileum and colon mucosal scrapings using the repeated bead-beating plus column method [15], with only a few changes [16]. Briefly, about 250 mg of each sample were suspended in 1 mL of lysis buffer (500 mM NaCl, 50 mM Tris-HCl pH 8, 50 mM EDTA, 4% SDS) and bead-beaten three times in a FastPrep-24 Instrument (MP Biomedicals, Irvine, CA, USA) at 5.5 movements per sec for 1 min, in the presence of four 3-mm glass beads and 0.5 g of 0.1-mm zirconia beads (BioSpec Products, Bartlesville, OK, USA). After 15-min incubation at 95 °C and centrifugation at full speed for 5 min to pellet raw materials, nucleic acids were precipitated by adding 10 M ammonium acetate and one volume of isopropanol, followed by incubation in ice. Pellets were washed with ethanol 70%, resuspended in TE buffer, and treated with 10 mg/mL DNase-free RNase at 37 °C for 15 min. Proteinase K treatment and column-based DNA purification were performed following the manufacturer’s instructions (QIAamp DNA Stool Mini Kit; QIAGEN, Hilden, Germany). Paired-end sequencing of the V3-V4 hypervariable region of the 16S rRNA gene was performed on an Illumina MiSeq platform, at IGA Technology Services (http://igatechnology.com/; Udine, Italy). Sequence reads were deposited in the MG-RAST database (https://www.mg-rast.org/linkin.cgi?project=mgp87784). Raw sequences were processed using a pipeline combining paired-end assembler for Illumina sequences (PANDAseq) [17] and Quantitative Insights Into Microbial Ecology (QIIME) [18]. High-quality reads were binned into Operational Taxonomic Units (OTUs) at 97% similarity using UCLUST [19]. Taxonomy was assigned using the Ribosomal Database Project (RDP) classifier against Greengenes database (May 2013 release). Chimeric and singleton OTUs were discarded. Alpha rarefaction was performed using the Faith’s phylogenetic diversity, observed OTUs, and Shannon index metrics. Beta diversity was estimated by computing weighted and unweighted UniFrac distances.

### 2.5. Metabolomics

Two different analytical approaches were adopted to describe the metabolic profile of fecal samples, namely proton nuclear magnetic resonance (^1^H-NMR) for the liquid fraction and gas chromatography-mass spectrometry (GC-MS) for volatile compounds. ^1^H-NMR analysis was then also extended to ileum and colon content.

Fecal samples and samples from feces and colon were prepared for ^1^H-NMR analysis by following Bryszewska et al. [12]. In order to apply NMR as a quantitative technique [20], the recycle delay was set to 5 s, while keeping into consideration the longitudinal relaxation time of the protons under investigation. The signals were assigned by comparing their chemical shift and multiplicity with Chenomx software data bank (Chenomx Inc., Edmonton, AB, Canada, ver 8.1) and literature [21]. ^1^H-NMR spectra baseline was adjusted as described by Bryszewska et al. [12]. Differences in soluble solids content among samples was taken into consideration by probabilistic quotient normalization [22].

Solid-phase microextraction–gas chromatography–mass spectrometry (SPME–GC–MS) analysis was used to detect volatile metabolites in feces. Sampling, GC-MS conditions, and data processing were carried out according to Taneyo Saa et al. [23]. Partial least squares Discriminant Analysis (PLS-DA) was used to reduce data dimensionality and the plots of the first 2 x-variates allowed the visualization of time separation in each group according to Dejean et al. [24]. Random forest (RF) was used as feature selection technique to identify the molecules that are accountable for discriminating times (t0 and t30) within each diet and diets after the intervention (t30). RF-feature selection was obtained by calculating Importance Scores, i.e., the mean decrease in accuracy as reported by Enot et al. [25]. Pearson correlation coefficients between the 10 most important molecules identified by RF were calculated. Variables with a correlation coefficient greater than 0.7 were considered as belonging to a group that is indicative of different breakdown products during the diet.

### 2.6. Statistics

Data of blood analyses and body weight form Tr1 and Tr2 were analyzed by MedCalc Statistical Software version 16.4.3 (MedCalc Software bvba, Ostend, Belgium; https://www.medcalc.org; 2016). Specifically, in Tr1, for glucose and insulin measures, the data were normalized and analyzed by the non-parametric Kruskal–Wallis test. When the test was positive (*p* value < 0.05), post-hoc analysis according to Conover, 1999 was performed [26]. In Tr2, CBC data were analyzed by Wilcoxon test for paired samples while body weight data were analyzed with ANOVA test for repeated measurements. For microbiota and metabolome, all statistical analyses, including principal coordinate analysis (PCoA), ANOVA, and non-parametric tests (Wilcoxon test for paired or unpaired data as needed), were performed in R (http://www.R-project.org). *p* values were corrected for multiple comparisons using the Benjamini–Hochberg method when appropriate. A corrected *p* value < 0.05 was considered as statistically significant unless otherwise stated. In the water-soluble metabolome, differences between two groups of samples were analyzed by Wilcoxon non-parametric test, while differences that were induced by wheat and einkorn diets at t30 in the ileum-colon-feces trends were looked for by ANOVA test for repeated measurements. Robust principal component analysis (RPCA) models were calculated according to Hubert et al. [27]. For fecal samples, this was done on the t30–t0 differences, to consider the paired nature of the data.

### 2.7. Data Availability

The datasets generated and analyzed during the current study are available from the corresponding authors on reasonable request.

## 3. Results

### 3.1. Postprandial Glycemic and Insulinemic Response

**Figure 1:**
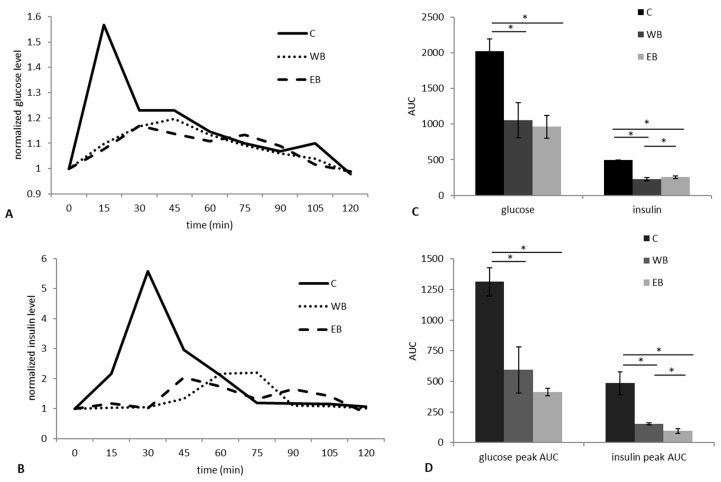
Postprandial glucose and insulin responses after einkorn (EB) or wheat (WB) bread consumption in the pig model (n = 4). (**A**) Glucose and (**B**) insulin trend waves among diets (fold of increase with respect to time 0). Area under the curve (AUC) for the entire curve (**C**) and for the first peak (**D**), in the three groups (EB, 50 g of einkorn bread; WB, 50 g of wheat bread; C (control), 50 g of glucose as a reference food). * for *p* value < 0.05, Kruskal–Wallis test.

In a first study trial (Tr1), we determined the postprandial glucose and insulin blood levels after einkorn (EB) or wheat (WB) bread consumption in comparison to the reference food (glucose), by sampling blood from the pig jugular vein up to 2 h after administration (Supplementary Table S2). Postprandial glucose levels differed between WB and EB at minute 15 and 105, with lower values for EB (*p* value = 0.0072 and *p* value = 0.012, respectively; Kruskal–Wallis test). At minute 15, the postprandial insulin level after WB administration was slightly higher (*p* value = 0.012; Kruskal–Wallis test) than EB. Glucose and insulin trends are represented in Figure 1A,B. The shape of the glycemic and insulinemic response waves differed between WB and EB, with a biphasic shape for EB, for both glucose and insulin. For each experimental bread as well as for the reference food, the area under the curve (AUC), the glycemic (GI), and insulinemic (II) index and the glycemic (GL) and insulinemic (IL) load were calculated (Figure 1C). GI was 52.1 ± 12.1 and 47.5 ± 7.7 for WB and EB, respectively. GL was calculated taking into account the percentage of carbohydrate in experimental breads (WB = 60.4%; EB = 59.9%) and it was 31.5 ± 7.3 and 28.4 ± 4.6 for WB and EB, respectively. No differences were observed between the two diets for GI and GL, but both differed from the reference food (*p* value = 0.018; Kruskal-Wallis test). II and IL were 46.2 ± 4.3 and 27.9 ± 2.6 for WB, and 51.7 ± 3.3 and 30.9 ± 2 for EB. The EB consumption resulted in a postprandial II and IL higher than WB (*p* value = 0.01; Kruskal–Wallis test). According to the peak analysis, the AUC of glucose and insulin first peaks that were produced by EB were lower than those produced by WB, but the difference was statistically significant only for insulin (*p* value = 0.024; Kruskal-Wallis test) (Figure 1D).

### 3.2. Complete Blood Count (CBC), Fecal Score and Body Weight

In a second study trial (Tr2), we investigated the impact of a 30-day nutritional intervention with EB or WB on the animal physiology and intestinal ecosystem. Data on complete blood count (CBC) and body weight are reported in Supplementary Table S3 and Supplementary Figure S1. No significant differences were observed in body increment between the two diets at 7, 15, and 30 days. The body weight increased for both diets from t0 to t15 (*p* value < 0.01; ANOVA for repeated measurements) and reached a plateau phase from t15 up to the end of the trial. Regarding CBC, levels of hematocrit, hemoglobin, red blood cells (RBCs), and reticulocytes increased during the trial from t0 to t30 in both groups, falling within the physiological reference intervals for the swine species [28]. Inflammation and immunological blood variables were not altered, even though in the first 10 days of the trial, both groups showed mild self-limiting diarrhea, which is likely due to the diet change (Supplementary Table S4). Specifically, the feces were watery during the first week and became fluffy during the second week. A score was then calculated based on stool consistency. According to the fecal score distribution, watery feces were no longer detected since t7 and t15 in the EB and WB group, respectively. In both diet groups, the feces of all pigs were normal in the second part of the trial (t15–t30).

### 3.3. Gut Microbiota Response to Nutritional Intervention

**Figure 2:**
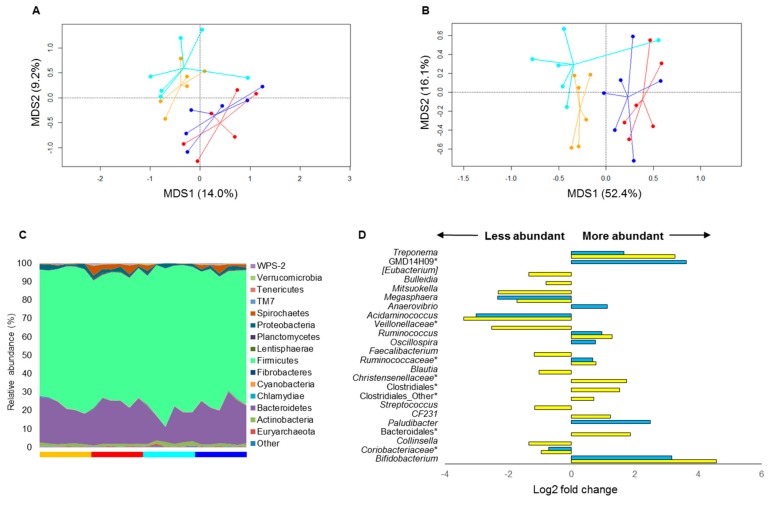
Modulation of the swine fecal microbiota after einkorn vs. wheat bread-based intervention. Principal Coordinate Analysis (PCoA) of unweighted (**A**) and weighted (**B**) UniFrac distances between the microbial profiles of pigs before and after 30-day consumption of einkorn (cyan, t0; blue, t30) or wheat bread (orange, t0; red, t30). A significant separation between t0 and t30 samples was found for both groups, according to both metrics (*p* value ≤ 0.003, permutation test with pseudo-F ratios). (**C**) Relative abundance of phylum-level taxa in the fecal microbiota of einkorn vs. wheat bread-fed pigs. Bars below the area chart are colored according to the diet group and time point, as in (**A**). (**D**) Genus-level signatures of response to nutritional interventions, shown as Log2 fold changes between t30 and t0 samples for the einkorn (light blue) or wheat (yellow) bread group. *, unclassified Operational Taxonomic Units (OTUs) reported at higher taxonomic level. *p* value < 0.05, Wilcoxon test.

Stool samples collected at 0, 15, and 30 days of nutritional intervention (Tr2) were subjected to next-generation sequencing of the 16S rRNA gene. A total of 351,786 high-quality reads (mean per sample, 9772; range, 7207–11,734) were obtained and analyzed. Reads were clustered into 16,236 OTUs at 97% similarity. According to the Shannon index, the alpha diversity significantly increased after 30 days of diet in both pig groups, regardless of the type of bread administered (t0 vs. t30, WB, 7.07 vs. 7.58; EB, 6.93 vs. 7.58; *p* value ≤ 0.04, Wilcoxon test). Similarly, weighted and unweighted UniFrac distance ordination showed separation between t0 and t30 samples for both the EB and WB group (*p* value ≤ 0.003, permutation test with pseudo-F ratios) (Figure 2A,B). At phylum level, the swine fecal microbiota was dominated almost exclusively by Firmicutes (mean relative abundance at t0, 76.24%) and Bacteroidetes (18.48%), with Actinobacteria (1.67%) and Proteobacteria (1.68%) as subdominant components (Figure 2C). Interestingly, both diets resulted in similar increases in the proportion of Spirochaetes, Lentisphaerae, and Tenericutes (*p* value ≤ 0.04, Wilcoxon test), but only in the EB group there was a significant modulation of the dominant microbiota fraction, with increased representation of Bacteroidetes and corresponding depletion of Firmicutes (Firmicutes to Bacteroidetes ratio: t0 vs. t30, 5.58 vs. 3.25; *p* value = 0.04) (Figure 2C). It is worth noting that this variation was already appreciable at t15 (Firmicutes to Bacteroidetes ratio, 2.58; *p* value = 0.002) (Supplementary Figure S2). On the other hand, only the WB consumption led to an increase in the relative abundance of Verrucomicrobia (*p* value = 0.01) (Figure 2C). Genus-level taxonomic comparisons confirmed the existence of both common and unique signatures in response to nutritional interventions (*p* value < 0.05) (Figure 2D). Among the shared features, it is worth noting that both diets, already at t15, resulted in increased abundance of *Treponema*, *Ruminococcaceae* species (including *R. bromii*), and bifidobacteria, and diminished proportions of *Coriobacteriaceae* members, *Acidaminococcus,* and *Megasphaera*. Interestingly, only the EB consumption favored the enrichment of the following minor components: *Oscillospira*, *Anaerovibrio*, *Paludibacter*, unclassified members in the GMD14H09 order (Deltaproteobacteria class), and *Blautia producta*. Conversely, the abundances of *Blautia* spp. were significantly reduced in the fecal microbiota of WB-fed pigs, as well as those of other SCFA producers, such as *Faecalibacterium* and *Dorea formicigenerans*. The WB consumption also led to the reduction of *Collinsella*, *Streptococcus*, *Veillonellaceae* (*Mitsuokella* and unknown genera), and *Erysipelotrichaceae* (*Bulleidia* and [*Eubacterium*]) genera. On the other hand, it favored the enrichment of unclassified members within the Bacteroidales and Clostridiales orders. Finally, yet importantly, after 30 days of nutritional intervention, the fecal microbiota of pigs administered with EB differed from that of pigs fed with WB for a diminished relative abundance of members of the Bacteroidales family S24-7 (EB vs. WB group, 3.24% vs. 5.47%; *p* value = 0.04).

### 3.4. Mucosa-Associated Microbiota from Ileal and Colonic Compartments

**Figure 3:**
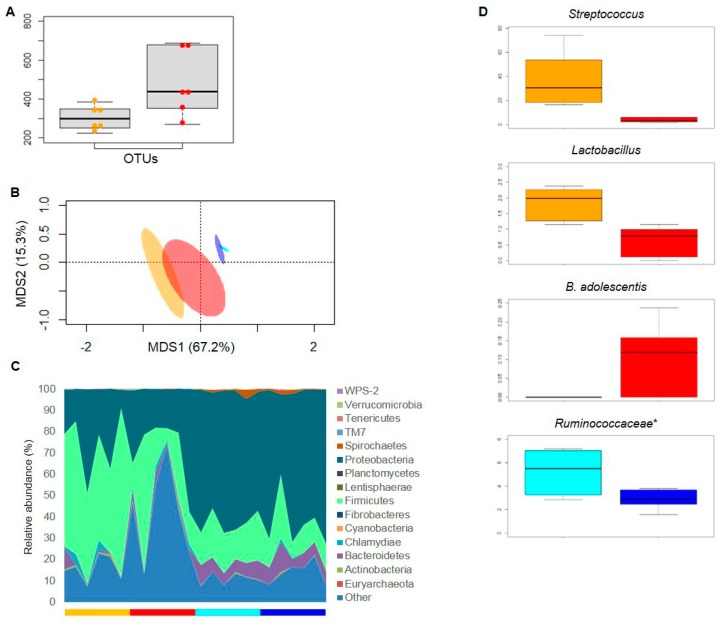
Mucosa-associated microbiota from ileum and colon after einkorn vs. wheat bread-based intervention. (**A**) Box plots showing the distribution of alpha diversity values, according to the observed OTU metrics. Samples are identified with colored dots within boxes (orange: ileal mucosa from wheat bread (WB)-fed pigs; red, ileal mucosa from einkorn bread (EB)-fed pigs). A significant difference between diet groups was found (*p*-value = 0.02, Wilcoxon test). (**B**) Principal Coordinate Analysis (PCoA) of weighted UniFrac distances between the mucosal microbial profiles of pigs after 30-day consumption of EB (ileum, red; colon, blue) or WB (ileum, orange; colon, cyan). Ellipses include 99% confidence area based on the standard error of the weighted average of sample coordinates. Only in the ileum compartment, the mucosa-associated microbiota fractions clustered in distinct groups according to diet (*p*-value = 0.04, permutation test with pseudo-F ratios). (**C**) Relative abundance of phylum-level taxa in the ileal or colonic mucosal microbiota of EB- vs. WB-fed pigs. Bars that are below the area chart are colored according to diet and intestinal compartment, as in (**B**). (**D**) Box plots showing the distribution of the relative abundance values of discriminant taxa between the EB and WB group, at the ileum or colon level. Same color code as in (**B**). *, unclassified OTU reported at higher taxonomic level. *p* value < 0.05, Wilcoxon test.

Mucosal scrapings from ileum and colon were collected after nutritional intervention and subjected to 16S rRNA gene-based next-generation sequencing, as reported above for fecal samples. A total of 158,871 high-quality reads (mean per sample, 6620; range, 3315–9913) were obtained and clustered into 10,560 OTUs at 97% similarity. According to the observed OTU metrics, the ileal mucosa-associated microbiota after 30 days of EB consumption was more biodiverse than after WB administration (*p* value = 0.02, Wilcoxon test) (Figure 3A). Similarly, only at the ileum level and only in the weighted UniFrac distance-based ordination space, the mucosal microbial fractions clustered in distinct groups according to diet (*p* value = 0.04, permutation test with pseudo-F ratios) (Figure 3B). Unlike feces, the phylum-level microbiota structure of the ileal mucosa was dominated by Firmicutes (mean relative abundance, 39.28%) and Proteobacteria (26.77%), whose proportions were inverted and even more differentiated at the colonic mucosa level (Firmicutes, 16.80%; Proteobacteria, 61.55%) (Figure 3C). In both intestinal compartments, Bacteroidetes was the main sub-dominant phylum, followed mostly by Chlamydiae in the ileum and Spirochaetes in the colon. Interestingly, when compared to WB-eating pigs, the ileal mucosa-associated microbiota fraction of pigs fed with EB was largely enriched in unclassified microbial members (EB vs. WB group, 42.49% vs. 15.58%; *p* value = 0.02, Wilcoxon test), while profoundly depleted in Firmicutes (24.37% vs. 54.20%; *p* value = 0.04) (Figure 3C). At the genus level, this difference was mainly attributable to an underrepresentation of *Streptoccoccus* (mostly the *S. alactolyticus* species) and *Lactobacillus* (mostly *L. ruminis*) in the EB as compared to the WB group. It is worth noting that, although largely subdominant, the species *Bifidobacterium adolescentis* was detected in the ileal mucosal fraction only after EB administration. At the colonic mucosa level, the consumption of EB resulted in a reduced abundance of unknown genera in the *Ruminococcaceae* family, when compared to the WB-based diet (Figure 3D).

### 3.5. Metabolomics on Water Soluble Fraction

**Figure 4:**
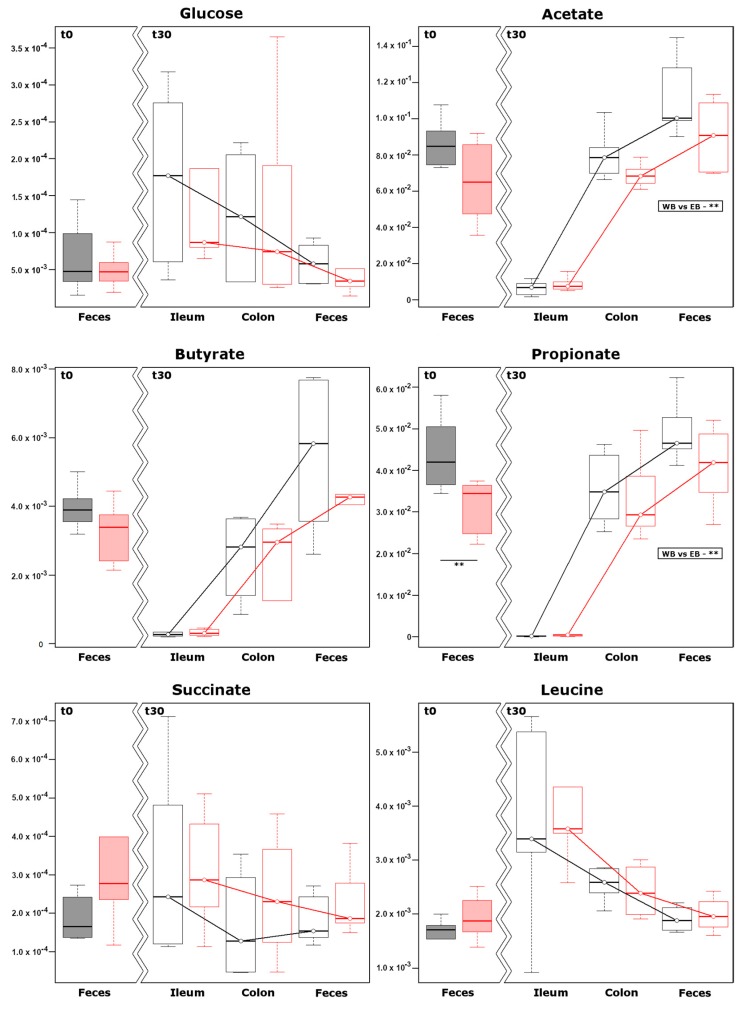
Concentration of glucose, short-chain fatty acids (SCFAs) (acetate, butyrate, and propionate), succinate and leucine along the pig intestine. Boxplots show, for wheat (black) and einkorn (red) bread-based diets, the concentration (mmol/L) of the metabolites in feces, at t0 and t30, and in ileum and colon contents, at t30. The inserts highlight the trends at t30 significantly affected by the diets, while significance of the differences at t0 or at t30 for feces are reported over the corresponding boxes. ** *p* value < 0.05.

**Figure 5:**
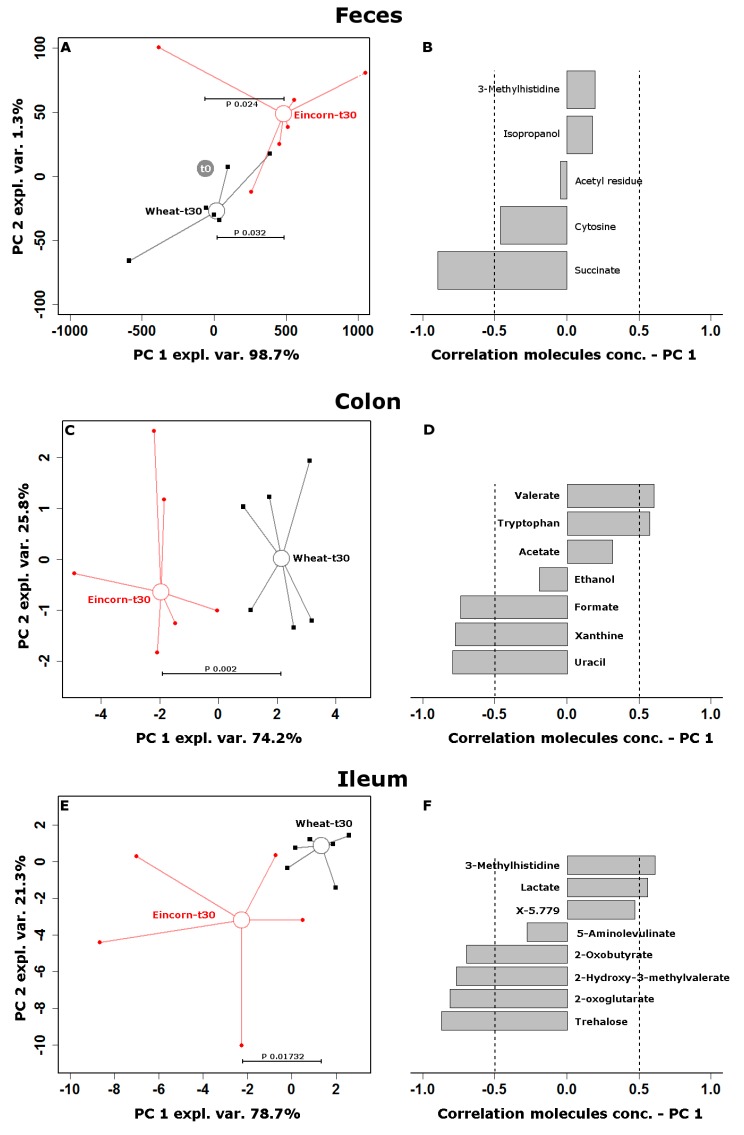
Metabolomics signatures along the pig intestine, associated with einkorn or wheat bread-based intervention. Robust principal component analysis (RPCA) models were calculated on the concentrations of significantly different molecules between the wheat (WB) and einkorn bread (EB) groups, as outlined in Supplementary Tables S5–S7, centered and scaled to unity variance. Panels (**A**,**C**,**E**) show the corresponding score plots, with samples from the WB group represented by black squares and samples from the EB group by red circles. The samples are connected to their median values by lines. Panels (**B**,**D**,**F**) show the corresponding correlation between molecules concentrations and their importance along PC 1, with dashed lines evidencing correlations lower than −0.5 and higher than 0.5. In panel (**A**), the samples at t0 are superimposed because their metabolome has been subtracted to the one of each sample from the same subject to consider the paired nature of the data.

In order to observe the effects of the two tested diets from a metabolomics perspective, the water-soluble fractions of ileum and colon contents and the feces were studied by proton nuclear magnetic resonance (^1^H-NMR). Firstly, we specifically focused on glucose, SCFAs (i.e., acetate, propionate and butyrate), succinate and leucine, whose concentration are known to be strictly related to insulin sensitivity [29,30,31,32,33] (Figure 4). Glucose, succinate and leucine showed decreasing concentrations along the intestinal tract for both diets. Within such a decreasing trend, the WB diet systematically led to a higher concentration of glucose and a slightly lower concentration of succinate at each time point when compared to the EB diet. SCFAs showed marked increases along the intestinal tract for both diets, with the WB diet associated with higher increases of acetate and propionate when compared to EB. As a second step of the metabolomics investigation, we broadened our view by employing ^1^H-NMR for an untargeted metabolomics approach. Seventy molecules could be quantified, pertaining to the chemical groups of monomeric carbohydrates, amino acids, SCFAs, and organic acids. Fifteen more molecules could be quantified, with a partially unclear structure, and therefore denoted with the location in the NMR spectrum, expressed in ppm. To evidence the peculiar effects of the two diets, we firstly focused on feces, by calculating the t0–t30 variation for each molecule and selecting those showing variations that differed between the two diets with a confidence higher than 90% (Supplementary Table S5). The five molecules so highlighted served as a base for an RPCA model. In the corresponding score plot (Figure 5A), PC 1, representing 98.7% of the total variance among samples, summarized the t0–t30 variation, with samples pertaining to the EB group at t30 being characterized by the highest scores, and samples pertaining to the WB group at t30 characterized by scores that are very close to those of samples at t0. The relative position on the two groups of samples at t30 was therefore significantly different (*p* < 0.05), but only the EB group significantly differed from samples at t0 (*p* < 0.05). The correlation plot (Figure 5B) evidenced that the most relevant molecule in the underlying structure of the data was succinate. Succinate was the only quantified molecule with a correlation between concentration and importance over PC 1 higher than 0.5. Univariate comparisons between the samples that were collected from the colon and ileum at t30 offered further opportunities to highlight the peculiarities of the two diets. The colon samples from the EB group differed from those of the WB group in the concentration of seven molecules (Supplementary Table S6). An RPCA model based on these molecules was able to clearly discriminate (*p* < 0.01) the samples from the two groups (Figure 5C), with valerate and tryptophan being more concentrated in the WB group, and formate, uracil, and xanthine more concentrated in the EB group (Figure 5D). A parallel analysis of ileum contents showed eight molecules to differ in concentration between the two groups (Figure 5E,F; Supplementary Table S7), with lactate, 3-methylhistidine, and molecule X-5.779 being more concentrated in the WB group, and 2-hydroxy-3-methylvalerate, trehalose, 2-oxoglutarate, 2-oxobutyrate, and 5-aminolevulinate more concentrated in the EB group.

### 3.6. Metabolomics on Volatile Fraction

**Figure 6:**
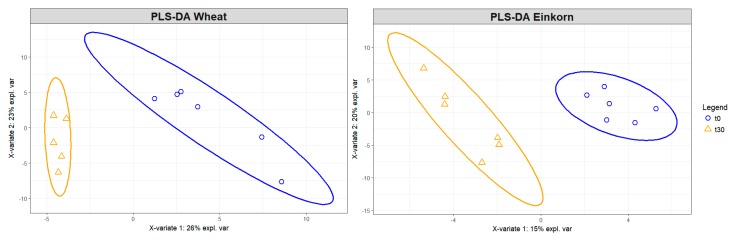
Distribution of fecal volatile molecules, analyzed by gas chromatography-mass spectrometry (GC-MS), before (t0) and after (t30) einkorn (EB) vs. wheat (WB) bread-based dietary intervention.

**Figure 7:**
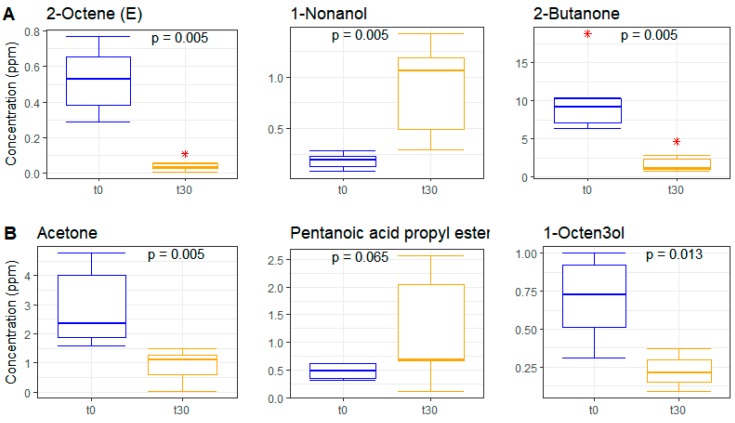
Boxplots showing the concentrations of the main fecal volatile molecules, analyzed by GC-MS, discriminating between t0 (blue) and t30 (orange) in the einkorn (EB; Figure 7**A**) and wheat (WB; Figure 7**B**) bread-based diet groups, as determined through Random Forest. *, outliers. *p* values were determined by Wilcoxon test.

**Figure 8:**
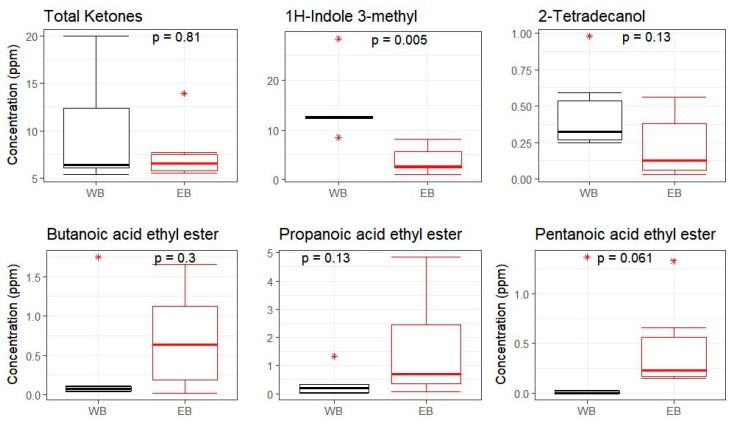
Boxplots showing the concentrations of the main fecal volatile molecules, analyzed by GC-MS, discriminating between the wheat (WB) and einkorn (EB) bread-based diet groups at the end of the intervention (t30), as determined through Random Forest. *, outliers. *p* values were determined by Wilcoxon test.

To describe the effects of the two experimental breads (WB and EB) on the fecal volatile metabolite profiles, around 90 volatile molecules were analyzed by solid-phase microextraction–gas chromatography–mass spectrometry (SPME-GC-MS) in fecal samples before and after the 30-day dietary intervention. Figure 6 shows the distribution of volatilomes before and after intervention. The two diets had an opposite effect on metabolite diversity: specifically, the WB diet led to reduced metabolite distribution while the EB diet resulted in increased metabolite diversity in experimental animals. The main metabolite changes of volatile molecules resulting from the Random Forest (RF) feature selection and subsequent Pearson correlation test are reported in Figure 7. Specifically, the WB diet determined a decrease in 2-octene and 2-butanone (*p* value = 0.005 for both molecules; Wilcoxon test) and an increase in 1-nonanol concentrations (*p* value = 0.005). On the other hand, the EB diet determined a decrease in acetone and 1-octen3-ol (*p* value = 0.005 and 0.013, respectively), and an increase in pentanoic acid propyl ester (*p* value = 0.065) (Figure 7). The out-of-bag (OOB) error rate was 0% for WB and 16.67% for EB. Interestingly, among the 10 most important molecules that are responsible for discriminating between t0 and t30, the WB diet was characterized by the presence of four ketones, whereas for the EB diet the ketones were six (Supplementary Figure S3). Among the 10 molecules, identified by RF, discriminating between WB and EB at the end of the trial (i.e., t30), two molecules, 1H-indole-3-methyl and 2-tetradecanol, showed a correlation greater than 0.7 (Pearson correlation showed in Supplementary Figure S4). The concentration of both molecules was higher after the WB diet as compared to EB, but only for 1H-indole 3-methyl the difference was significant, with a *p* value = 0.005 (Figure 8).

## 4. Discussion

The aim of the present study was to investigate in the pig model the impact of wheat (WB) and einkorn (EB) breads on the microbiota and metabolome structure, both in feces and along the intestinal tract, as well as on the glycemic and insulinemic response. According to our findings, both breads evocated an overall similar glycemic response, whereas the insulinemic response, in terms of insulin index and load, was higher after EB administration. Since the carbohydrate content of the breads was very similar, this difference could be attributed to the higher amount of protein in EB as compared to WB [34]. However, as previously highlighted by Abdel-Aal and Hucl [35], alongside with a higher protein content, einkorn products resulted in being relatively low in lysine and high in glutamic acid when compared to other wheat products. Moreover, the gliadin-to-glutenin ratio for einkorn proteins was 2:1, as compared to 1:1 for wheat, indicating some differences in the amino acid composition of these wheats. Other differences that were found in EB included a significantly higher content in lipids, tocols, lutein, and MUFAs and a lower one in SFAs and PUFAs [36]. Notwithstanding this, the analysis of insulin curves showed a lower peak for EB, suggesting a lower and more constant release of insulin over time, comparable to that of diets that promote a lasting sense of satiety [37]. This hypothesis seems to be confirmed by the lower glycemic peak induced by EB consumption (Figure 1D), which should evoke a less intense hypoglycemic response. Interestingly, the EB meal led to a biphasic shape for glycemic and insulinemic response waves. The biphasic shape was correlated with a lower risk of type 2 diabetes in both adolescents and adults [38], and recently, with lower cumulative incidence of type 1 diabetes [39]. In a study performed in a pig model of oral glucose tolerance test (OGTT), the prevalence of biphasic glycemic waves was shown to be correlated, as for humans, with female sex [40]. In our study, the animals enrolled in Tr1 were all females. We did not investigate the mechanism of action that led to the biphasic shape, but as the trial was designed to reduce the individual effect (latin squared design), we can reasonably assume that the biphasic shape was induced by EB. Further studies will be necessary to investigate this phenomenon. With respect to animal physiology, no significant alterations in blood parameters were observed over a 30-day nutritional intervention period (Tr2), except for a physiological increase in hematocrit, hemoglobin, red blood cells, and reticulocytes, which is consistent with normal animal development [28]. The body increment in the first two weeks of the study was linear for both diets, and was comparable to pig standards [41]. Conversely, it slowed down from t14 to t30 in both diet groups. Given that for experimental needs the animals received the same amount of food (1 kg die) for all 30 days of the trial, we argued that, from t14, that amount was no longer sufficient to sustain the high growth rate typical of commercial hybrid pigs. Moreover, from t0 to t14, the animals in both groups developed a moderate self-limiting diarrhea, most likely caused by the change of diet. It has indeed been reported that sudden dietary changes can be considered as a nutrition stressor that can challenge animal homeostasis [42], and that probiotics and anti-inflammatory diets can help to reduce the effects of this alteration [43]. Interestingly, in the EB group, watery feces were no longer observed from the second week of the trial, suggesting a better and faster recovery from dietary stress-related diarrhea, as compared to WB. These findings are consistent with our previous study in intestinal cell cultures, which highlighted an anti-inflammatory and protective effect for EB, but the hematological inflammatory indexes were not altered [4]. Probably, we were not able to detect an anti-inflammatory effect observing blood parameters, because the stress that was produced by the change of diet was very moderate and only affected the fecal aspect. A specific trial is needed to investigate the anti-inflammatory potential of einkorn-based products in the pig model.

When compared to the WB diet group, the fecal microbiota of EB-fed pigs was found to be enriched in several components that were regarded as health-promoting, including the well-known SCFA producers *Blautia* (acetogenic) and *Faecalibacterium* (butyrogenic), and *Oscillospira*. Interestingly, recent evidence points to *Oscillospira* as a key member of the gut microbiota, being negatively associated with inflammatory diseases and body mass index, probably through the production of butyrate from dietary or host glycoproteins [44]. In particular, relative *Oscillospira* abundance was found to increase with the switch to a diet rich in protein and fat [45], thus suggesting an interesting link with the protein and lipid profile of EB, which is worthy of further study. On the other hand, countless studies support the anti-inflammatory properties of *Faecalibacterium*, widely accepted as a biomarker of intestinal health [46], but also *Blautia*, which has been associated with decreased inflammation and improved outcomes in several clinical settings, including intestinal diseases, insulin resistance and obesity, and immunological disorders [47]. It is also worth noting that only the EB diet led to increased relative abundances of *Paludibacter*, a propionate and acetate-producing Bacteroidetes member [48], and *Anaerovibrio*, a swine commensal microorganism that is known for its lipase activity [49,50], which may support a direct role of this bacterial genus in the degradation of the high lipid content of EB, as compared to WB. The fecal microbiota of EB-fed pigs also showed a decreased relative abundance of the Bacteroidales family S24-7, which includes lipopolysaccharide (LPS)-producing members with a still unclear role in host physiology, but proven to have the capacity for opportunistic infection under appropriate conditions [51,52]. Not least, at the ileum level, the mucosa-associated microbiota fraction after 30-day EB intervention showed increased biodiversity, a community measure proposed as an indicator of eubiosis-associated health [53]. It is thus tempting to speculate that the consumption of EB could modulate the intestinal microbial profile towards a configuration that is less prone to inflammation, specifically enriched in microbes potentially contributing to maintaining the host homeostasis.

There is increasing evidence that molecules present in the intestinal lumen due to release from food or produced by the intestinal microbiota may play an active role in glucose homeostasis. In particular, the glucose available in the intestine may be a key factor of the central enhancement of insulin sensitivity for the whole body [30]. SCFAs, with specific reference to propionate, acetate, and butyrate, may act as glucose homeostasis regulators both when reaching the liver [31] and when produced in the gut lumen [32]. In addition, succinate and leucine produced by the microbiota have been indicated as potential improvers of glucose homeostasis, in particular succinate is able to induce intestinal gluconeogenesis serving as substrate [29,33]. To understand which of these known mechanisms was possibly active in the present study, an investigation by means of ^1^H-NMR was targeted to the aforementioned molecules, followed by an untargeted observation of the ileum, colon, and fecal metabolome. This two-step approach highlighted the key role of succinate as the fecal molecule that mostly contributed to the samples pattern. While being equally concentrated in the feces of the animals at t0, succinate was indeed found to be significantly lowered at t30 after the EB diet, suggesting that this molecule was absorbed more efficiently, with potential positive effects on glucose homeostasis. We cannot even rule out a higher rate of succinate utilization by intestinal bacteria [54], as suggested by the greater relative abundance of Bacteroidetes members, observed only after the EB diet, which are known to utilize the succinate pathway as the major route for propionate formation [55]. Moreover, the RPCA model revealed that the EB diet had been able to significantly impact on the animals’ fecal metabolome, while the WB one could not. Confirming such findings, the metabolomes of ileum and colon contents showed significant differences according to diet. Among the molecules mostly contributing to this difference, it is noteworthy to highlight the high importance of tryptophan, more concentrated in the WB group, which is known to play a key role in small intestine motor function as well as in a series of cascade events that are mediated by indolamine dioxygenase (IDO).

GC-MS analysis reported a quite different metabolic fingerprint for the two diets, with higher metabolite diversity in the fecal samples of pigs that were fed with EB as compared to WB. This is in agreement with previous findings on ancient grains dealing with their effects on the gut microbiota and their metabolites [23]. Specifically, when compared to WB, the volatile metabolome of EB-fed pigs showed a reduced abundance of some alcohols, including 2-tetradecanol and 1-octen-3-ol, and 1H-indole-3-methyl (i.e., skatole), which has been reported to cause tissue damage. Indeed, indole appears as a bacterial metabolite that is produced from tryptophan, highly concentrated in WB diet, as reported above. Higher concentrations of such metabolite may represent a counteraction towards detrimental effects of LPS in the liver upon WB diet, as recently reported by literature [56]. The reduced presence of alcohols could suggest a reduced production and therefore a reduced risk of endotoxemia, as discussed elsewhere [57,58]. On the other hand, skatole levels have been found to decrease in the hindgut and feces of pigs that were fed with increasing levels of fermentable carbohydrates, leading to increasing SCFA amounts and pH decline, most likely as a result of a reduced presence of skatole-producing bacteria and/or an altered tryptophan metabolism towards indole production [59]. In this regard, it is worth mentioning that *Lactobacillus*, one of the first bacterial genera identified as a skatole producer [60], was found to be largely underrepresented in the mucosa-associated microbiota of pigs that were fed with EB as compared to WB. Among the most discriminating volatile molecules, we also found an increased amount of pentanoic acid propyl ester, as well as butanoic and propanoic acid ethyl esters in the EB-based diet group. In general, the esterification by bacterial metabolism is adopted in the liver, pancreas, and intestine to remove toxic concentrations of metabolites, including SCFAs [61], which makes their presence a clear consequence of their previous production.

## 5. Conclusions

In summary, through the pig model we demonstrated a beneficial impact of EB on several aspects of the host physiology, including insulin release, fecal consistency, and microbiota and metabolome profiles, both in feces and intestinal contents. According to our findings, the consumption of EB could reduce the AUC of the first insulin peak, thus prolonging the sense of satiety. Moreover, it could modulate the intestinal ecosystem, at both the compositional and metabolic scale, towards a configuration specifically enriched in health-promoting bacteria and showing distinct metabolic signatures potentially contributing to maintaining the host homeostasis. The use of the pig model allowed, unlike in clinical human trials, the sampling of the mucosa and the content of the small intestine, thus widening the knowledge on the complexity of the food-microbiota-host interaction along the gastrointestinal tracts. The observed positive effects could be driven by the synergistic interaction of many factors, including, *inter alia*, the fermentation process, the food matrix, and the flour components, which result in gut-mediated effects. The evaluation of the beneficial effects of a real food is far more complex than using purified compounds, as a direct cause-effect relationship can seldom be ascribed to a single component. It is indeed foods, and not the single components, which create the diet, and exploring their complexity can better reflect their overall role on health. Although further studies and clinical trials are needed, the results that are herein reported represent a first contribution to unravel the anti-inflammatory potential of einkorn-based foods.

## Supplementary Materials

The following are available online at http://www.mdpi.com/2072-6643/11/1/16/s1. Supplementary Table S1. Standard Swine Diet composition (Big 30–80 by CESAC s.c.a); Supplementary Table S2. Postprandial glucose blood levels and insulin plasma levels after einkorn (EB) or wheat (WB) bread consumption in the pig model (n = 4). EB, 50 g of einkorn bread; WB, 50 g of wheat bread; C, 50 g of glucose as a reference food. Values are expressed as mean ± SD. Different lowercase letters indicate significant differences between diets (*p* value < 0.05; Kruskal-Wallis test on normalized values); Supplementary Table S3. Complete Blood Count (CBC) and body weight of the pigs enrolled in the Trial 2 expressed as mean ± Standard Error of the Mean (SEM) in the two diet groups (WB, wheat bread, n = 6; EB, einkorn bread, n = 6). RBCs: red blood cells; WBCs: white blood cells. Different lowercase letters indicate significant differences within each group between t0 and t30 (*p* < 0.05, Wilcoxon test); Supplementary Table S4. Frequency distribution of the daily fecal score in the two diet groups (WB, wheat bread, n = 6; EB, einkorn bread, n = 6) during the four weeks of Trial 2. 1 = watery feces; 0.5 = fluffy feces; 0 = normal feces; Supplementary Table S5. Concentration (mM) expressed as mean ± standard deviation of molecules whose t0–t30 variation in feces was statistically different between the wheat (WB) and einkorn bread (EB) groups (*p* < 0.1). *p*-values were calculated by Wilcoxon test; Supplementary Table S6. Concentration (mM) expressed as mean ± standard deviation of molecules (*p* < 0.1) different between the wheat (WB) and einkorn bread (EB) groups in colon contents. *p*-values were calculated by Wilcoxon test; Supplementary Table S7. Concentration (mM) expressed as mean ± standard deviation of molecules (*p* < 0.1) different between the wheat (WB) and einkorn bread (EB) groups in ileum contents. *p*-values were calculated by Wilcoxon test; Supplementary Figure S1. Body weight increment following einkorn (EB, n = 6) vs wheat bread (WB, n = 6) administration. For both diets (WB in black and EB in red) the body weight incremented up to day 15 (t15) with a *p* value < 0.01 (ANOVA for repeated measurement) and then reached a plateau phase; Supplementary Figure S2. Firmicutes to Bacteroidetes ratio in the fecal microbiota of pigs fed with einkorn vs wheat bread. Box plots showing the distribution of the Firmicutes to Bacteroidetes ratio in the fecal microbiota of pigs before (t0) and after 15 (t15) and 30 (t30) days of consumption of wheat (WB) or einkorn bread (EB). A significant reduction in the ratio was found in the EB diet group, both at t15 and t30 (*p*-value ≤ 0.04, Wilcoxon test); Supplementary Figure S3. (**A**) Random Forest Classification of the 10 most important molecules responsible for discriminating between t0 and t30 in WB and EB diet. (**B**) Pearson correlation test among the identified molecules for WB and EB diet; Supplementary Figure S4. (**A**) Random Forest Classification of the 10 most important molecules responsible for discriminating between WB and EB diet at the end of the trail, t30. (**B**) Pearson correlation test among the identified molecules.
