# Supplementary material for: In Vivo Effects of Einkorn Wheat (Triticum monococcum) Bread on the Intestinal Microbiota, Metabolome, and on the Glycemic and Insulinemic Response in the Pig Model
Source: Nutrients. 2018 Dec 20;11(1):16. doi: 10.3390/nu11010016 (PMC6356388; doi:10.3390/nu11010016)
Supplement: Supplementary file 1 [file nutrients-11-00016-s001.pdf]

SUPPLEMENTARY INFORMATION

Supplementary Table S1. Standard Swine Diet composition (Big 30-80 by CESAC s.c.a).

**Components**	
Corn, soy flour, wheat bran, barley, white sorghum, calcium carbonate, vegetable oils and fats, sodium chloride	
**Analytical components %**	
Crude protein 16.50%, crude oils and fats 3.50%, crude cellulose 4.70%, crude ash 4.10%, calcium 0.50%, phosphorus 0.40%, sodium 0.10%, lysine 0.90%, methionine 0.30%	
Additives/kg	
Vitamins and pro-vitamins	Vit H 0.25 mg
Vit A E627 12500 UI	Compounds of trace elements
Vit D3 E671 2000 UI	E 4 cupric sulfate, pentahydrate 79 mg
Vit E 3700 50 mg	E 8 sodium selenite 0.55 mg
Vit B1 2.0 mg	E 6 zinc oxide 155 mg
Vit B2 4.0 mg	E1 ferrous sulfate monohydrate 456 mg
Vit B6 3.0 mg	E5 manganous oxide 129 mg
Vit B12 0.025 mg	E2 potassium iodide 2.0 mg
Vit K 2.5 mg	Antioxidants
Vit PP 25 mg	Ethoxyquin E324 34 mg
Pantothenic acid 12.5 mg	Butylated hydroxyanisole (BHA) E320 30 mg

Supplementary Table S2. Postprandial glucose blood levels and insulin plasma levels after einkorn (EB) or wheat (WB) bread consumption in the pig model (n=4). EB, 50 g of einkorn bread; WB, 50 g of wheat bread; C, 50 g of glucose as a reference food. Values are expressed as mean ± SD. Different lowercase letters indicate significant differences between diets (P value<0.05; Kruskal-Wallis test on normalized values).

**Time** (min)	**Glucose Blood level** (mg/dl)			**Insulin Plasma level** (μU/ml)		
	C	WB	EB	C	WB	EB
0	77.3±0.8	83.3±1.2	81.3±0.9	3.6±1.7	5.3±0.2	4.4±0.7
15	121±4[a]	91.5±1.5[b]	87.4±1.4[c]	7.8±1.6[a]	5.4±0.1[b]	5.1±0.4[c]
30	95±6	97.3±1	95±1.8	20.1±0.2[a]	5.5±0.2[b]	4.4±0.4[b]
45	95±10.2	99.8±3.1	92.5±1	10.7±9.6	7±0.2	8.9±1.4
60	88.5±8.4	94.5±9	90±2.6	7.6±5.6	11.4±0.4	7.6±0.4
75	85±0.7	90.9±5.2	92±2.6	4.3±0.2	11.6±0.4	5.7±0.4
90	82.5±1.6	88.3±5.1	88.5±2.4	4.2±0.9	5.8±0.1	7.2±0.8
105	85±2[a]	86.5±2.6[b]	82.5±1.6[c]	4.1±1.2	5.7±0.1	6.2±0.9
120	75.5±1.6[a]	81.9±1.7[b]	80.3±0.6[b]	3.8±1.2	5.0±0.3	3.7±0.3

Supplementary Table S3. Complete Blood Count (CBC) and body weight of the pigs enrolled in the Trial 2 expressed as mean ± Standard Error of the Mean (SEM) in the two diet groups (WB, wheat bread, n=6; EB, einkorn bread, n=6). RBCs: red blood cells; WBCs: white blood cells. Different lowercase letters indicate significant differences within each group between t0 and t30 ($p<0.05$, Wilcoxon test).

	WB		EB	
	t0	t30	t0	t30
Body weight (kg)	37.4 ± 1.5^a	51.4 ± 2.9^b	31.8 ± 1.6^a	46.1 ± 3.5^b
Haematocrit (%)	32.0 ± 0.9^a	35.4 ± 1.1^b	32.5 ± 0.7^a	36.2 ± 0.8^b
RBCs (10^6/mm^3)	6.0 ± 0.2^a	6.8 ± 0.2^b	6.5 ± 0.2^a	7.1 ± 0.5^b
Hemoglobin (%)	10.4 ± 0.5	11.7 ± 0.3	10.6 ± 0.2^a	11.7 ± 0.2^b
Reticulocytes (%)	1.1 ± 0.0^a	2.3 ± 0.4^b	1 ± 0.1^a	2.7 ± 0.1^b
WBCs (x 10^4/mm^3)	2 ± 0.1	1.7 ± 0.2	2.4 ± 0.3	2.1 ± 0.2
Monocytes (%)	2.5 ± 0.2	2.7 ± 0.1	3 ± 0.2	3.1 ± 0.1
Neutrophils (%)	43.8 ± 3.2	40.5 ± 3.7	47 ± 2.0	42 ± 5.0
Eosinophils (%)	1.5 ± 0.5	2.1 ± 0.4	2.7 ± 0.4	3 ± 0.4
Lymphocytes (%)	51.2 ± 3.1	53 ± 4.0	46.2 ± 1.8	51 ± 4.5

Supplementary Table S4. Frequency distribution of the daily fecal score in the two diet groups (WB, wheat bread, n=6; EB, einkorn bread, n=6) during the four weeks of Trial 2. 1=watery feces; 0.5=fluffy feces; 0=normal feces.

	**WB**				**EB**			
Fecal score	Week1	Week2	Week3	Week4	Week1	Week2	Week3	Week4
1	100%	14.3%	0%	0%	71.4%	0%	0%	0%
0.5	0%	28.6%	0%	0%	28.6%	14.3%	0%	0%
0	0%	57.1%	100%	100%	0%	85.7%	100%	100%

Supplementary Table S5. Concentration (mM) expressed as mean ± standard deviation of molecules whose t0-t30 variation in feces was statistically different between the wheat (WB) and einkorn bread (EB) groups (P < 0.1). P-values were calculated by Wilcoxon test.

	t0	t30			t30-t0		
	WB and EB	WB	EB	P	WB	EB	P
3-methylhistidine	7.80E-5±4.51E-5	7.52E-5±1.77E-5	1.08E-4±4.73E-5	0.180	-2.06E-5±4.34E-5	4.82E-5±5.93E-5	0.065
Cytosine	1.64E-4±5.02E-5	1.53E-4±4.08E-5	7.37E-5±3.91E-5	0.026	-1.79E-5±4.33E-5	-8.33E-5±6.71E-5	0.093
Succinate	2.91E-4±2.56E-4	1.80E-4±6.23E-5	2.27E-4±8.80E-5	0.240	-7.08E-6±6.21E-5	-1.69E-4±2.60E-4	0.065
Acetyl residue	3.01E-5±2.78E-5	5.00E-5±1.09E-5	2.45E-5±2.07E-5	0.065	2.73E-5±1.38E-5	-1.31E-5±4.64E-5	0.026
Isopropanol	1.97E-4±1.04E-4	2.28E-4±5.16E-5	1.54E-4±1.90E-5	0.015	4.78E-5±3.44E-5	-6.08E-5±1.47E-4	0.041

Supplementary Table S6. Concentration (mM) expressed as mean ± standard deviation of molecules (P < 0.1) different between the wheat (WB) and einkorn bread (EB) groups in colon contents. P-values were calculated by Wilcoxon test.

	WB	EB	P
Formate	4.36E-5±9.23E-6	6.17E-5±1.41E-5	0.041
Tryptophan	7.72E-5±8.48E-6	5.36E-5±2.97E-5	0.093
Uracil	6.30E-4±1.18E-4	7.96E-4±6.67E-5	0.041
Acetate	8.02E-2±1.33E-2	6.89E-2±6.20E-3	0.093
Ethanol	5.30E-4±3.47E-4	6.80E-4±1.02E-4	0.065
Xanthine	3.49E-4±5.76E-5	4.60E-4±5.19E-5	0.009
Valerate	1.91E-2±2.47E-3	1.48E-2±2.48E-3	0.041

Supplementary Table S7. Concentration (mM) expressed as mean ± standard deviation of molecules (P < 0.1) different between the wheat (WB) and einkorn bread (EB) groups in ileum contents. P-values were calculated by Wilcoxon test.

	WB	EB	P
3-methylhistidine	3.83E-5±8.54E-6	1.95E-5±3.83E-6	0.004
X-5.779	2.33E-5±1.78E-5	4.90E-6±1.93E-5	0.052
Trehalose	6.94E-5±8.17E-5	4.74E-4±4.31E-4	0.017
Lactate	8.89E-2±2.18E-2	3.72E-2±2.54E-2	0.009
2-oxoglutarate	3.04E-4±1.06E-4	4.95E-4±1.64E-4	0.052
5-aminolevulinate	-1.29E-6±1.11E-5	4.17E-5±5.48E-5	0.052
2-hydroxy-3-methylvalerate	1.07E-4±4.61E-5	1.95E-4±9.87E-5	0.082
2-oxobutyrate	1.60E-5±1.05E-5	6.99E-5±4.18E-5	0.030

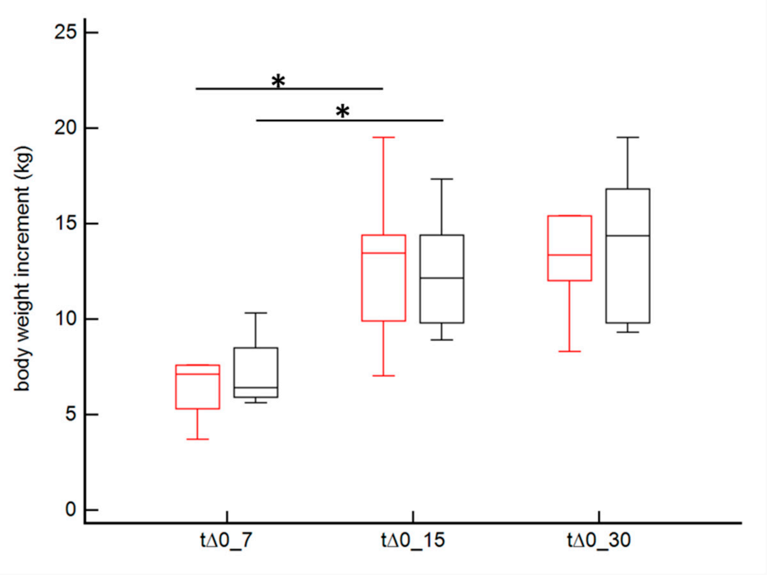

Supplementary Figure S1. Body weight increment following einkorn (EB, n=6) vs wheat bread (WB, n=6) administration. For both diets (WB in black and EB in red) the body weight incremented up to day 15 (t15) with a P value<0.01 (ANOVA for repeated measurement) and then reached a plateau phase.

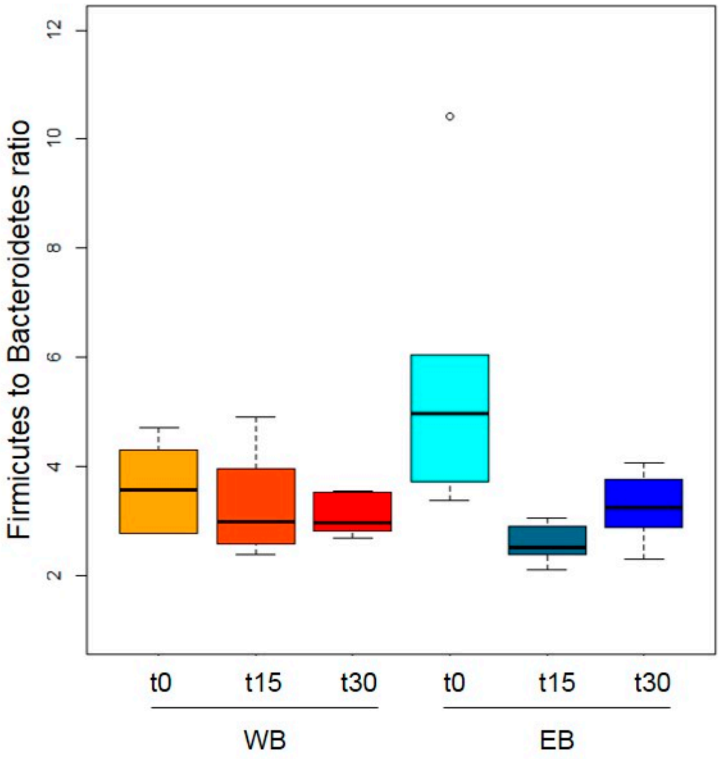

Supplementary Figure S2. Firmicutes to Bacteroidetes ratio in the fecal microbiota of pigs fed with einkorn vs wheat bread. Box plots showing the distribution of the Firmicutes to Bacteroidetes ratio in the fecal microbiota of pigs before (t0) and after 15 (t15) and 30 (t30) days of consumption of wheat (WB) or einkorn bread (EB). A significant reduction in the ratio was found in the EB diet group, both at t15 and t30 (P-value$\leq$0.04, Wilcoxon test).

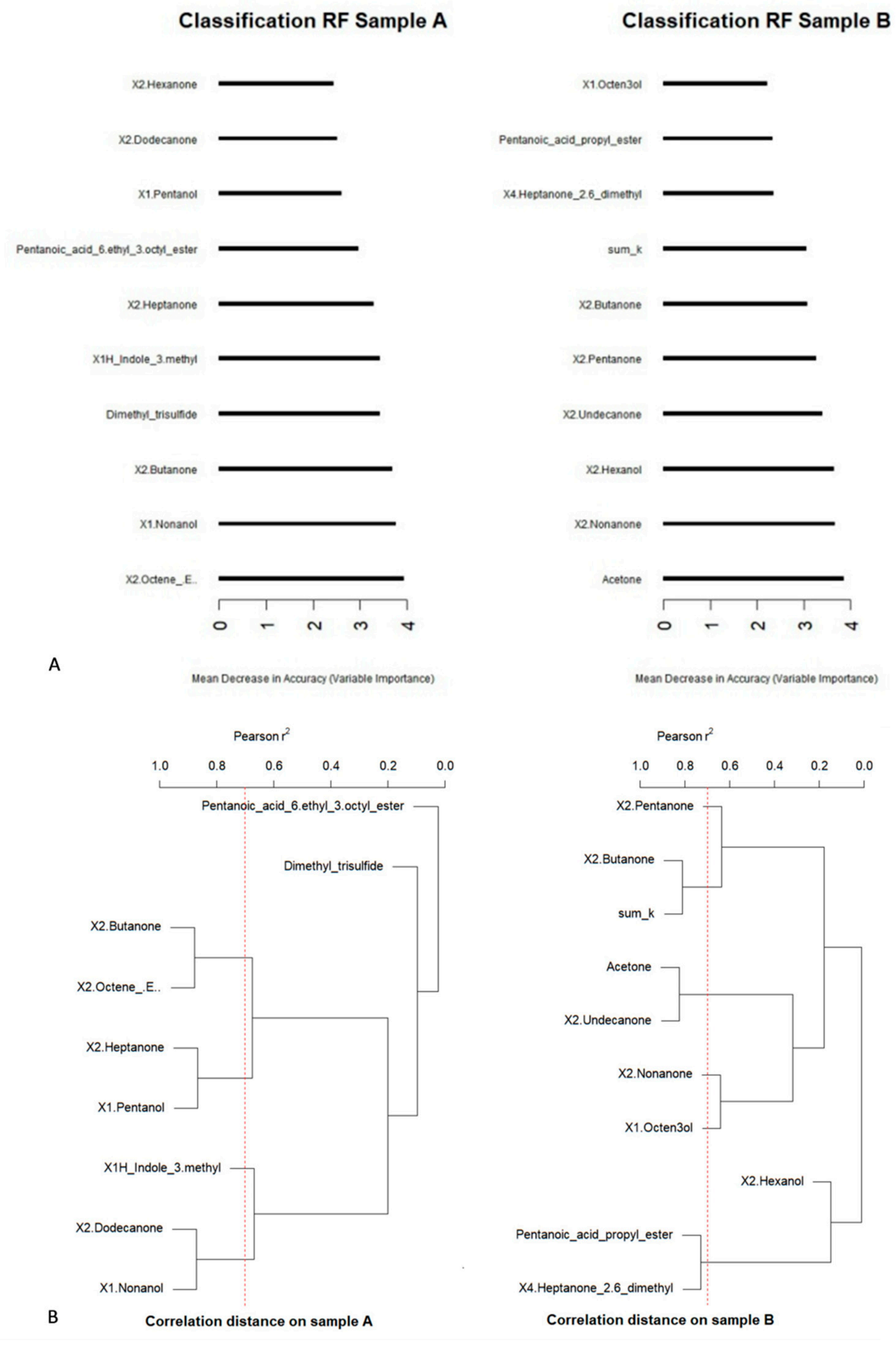

Supplementary Figure S3. (A) Random Forest Classification of the 10 most important molecules responsible for discriminating between t0 and t30 in WB and EB diet. (B) Pearson correlation test among the identified molecules for WB and EB diet.

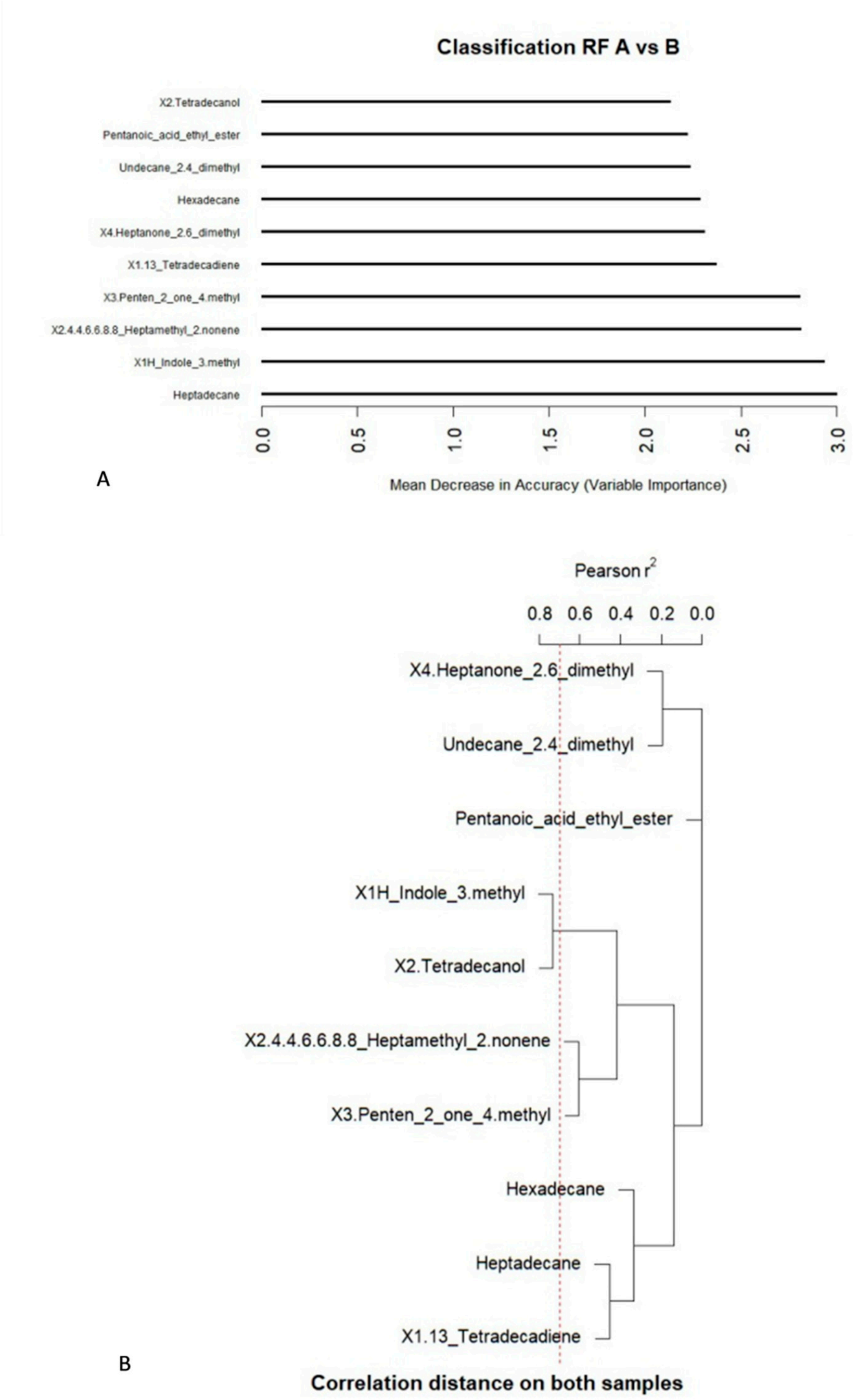

Supplementary Figure S4. (A) Random Forest Classification of the 10 most important molecules responsible for discriminating between WB and EB diet at the end of the trail, t30. (B) Pearson correlation test among the identified molecules.
